# Temporal Variation in the Microbiome of *Acropora* Coral Species Does Not Reflect Seasonality

**DOI:** 10.3389/fmicb.2019.01775

**Published:** 2019-08-16

**Authors:** Hannah E. Epstein, Hillary A. Smith, Neal E. Cantin, Veronique J. L. Mocellin, Gergely Torda, Madeleine J. H. van Oppen

**Affiliations:** ^1^ARC Centre of Excellence for Coral Reef Studies, James Cook University, Townsville, QLD, Australia; ^2^AIMS@JCU, James Cook University, Townsville, QLD, Australia; ^3^Australian Institute of Marine Science, Townsville, QLD, Australia; ^4^College of Science and Engineering, James Cook University, Townsville, QLD, Australia; ^5^School of BioSciences, University of Melbourne, Parkville, VIC, Australia

**Keywords:** coral microbiome, temporal variation, season, bacteria, Symbiodiniaceae, GBR, metabarcoding

## Abstract

The coral microbiome is known to fluctuate in response to environmental variation and has been suggested to vary seasonally. However, most studies to date, particularly studies on bacterial communities, have examined temporal variation over a time frame of less than 1 year, which is insufficient to establish if microbiome variations are indeed seasonal in nature. The present study focused on expanding our understanding of long-term variability in microbial community composition using two common coral species, *Acropora hyacinthus*, and *Acropora spathulata*, at two mid-shelf reefs on the Great Barrier Reef. By sampling over a 2-year time period, this study aimed to determine whether temporal variations reflect seasonal cycles. Community composition of both bacteria and Symbiodiniaceae was characterized through 16S rRNA gene and ITS2 rDNA metabarcoding. We observed significant variations in community composition of both bacteria and Symbiodiniaceae among time points for *A. hyacinthus* and *A. spathulata*. However, there was no evidence to suggest that temporal variations were cyclical in nature and represented seasonal variation. Clear evidence for differences in the microbial communities found between reefs suggests that reef location and coral species play a larger role than season in driving microbial community composition in corals. In order to identify the basis of temporal patterns in coral microbial community composition, future studies should employ longer time series of sampling at sufficient temporal resolution to identify the environmental correlates of microbiome variation.

## Introduction

Scleractinian corals are complex holobionts that host a high diversity and abundance of microbial symbionts that make up the coral microbiome (Blackall et al., 2015), some of which are essential to holobiont health, and function. Endosymbiotic microalgae of the family Symbiodiniaceae support coral growth and health by translocating carbon and other nutrients to the coral host (Lewis and Smith, 1971; Muscatine and Porter, 1977; Baker, 2001). Similarly, prokaryotes (i.e., bacteria and archaea) have been found to play a role in nutrient cycling, nitrogen fixation, and coral immunity (Rohwer and Kelley, 2004; Ritchie, 2006; Raina et al., 2009; Kimes et al., 2010; Bourne and Webster, 2013).

The coral microbiome is rarely static and its members can fluctuate as a result of changes in environmental conditions, or possibly due to host regulatory mechanisms (reviewed in Bourne et al., 2016). Symbiodiniaceae communities may fluctuate according to season (e.g., Chen et al., 2005; Ulstrup et al., 2008), but can remain stable through time in some coral species (e.g., Thornhill et al., 2006a,b). Additionally, significant stress events, such as high temperatures resulting in bleaching, can cause these microalgal symbionts to undergo shuffling (e.g., Berkelmans and van Oppen, 2006) or trigger the acquisition of novel strains from the environment (i.e., “switching”) (e.g., Boulotte et al., 2016). Further, the environment can modulate the initial uptake of Symbiodiniaceae, particularly in coral species that acquire these symbionts from the external environment during early development (LaJeunesse et al., 2004a). For prokaryote partners, variability in community composition is known to correlate with changing environmental conditions (reviewed in Thompson et al., 2015). Further, coral bacterial communities can vary geographically, where the same species can harbor vastly different communities of bacterial partners at different locations (e.g., Littman et al., 2009; Lee et al., 2012; Hernandez-Agreda et al., 2016). However, some bacterial members have been found consistently across all or most colonies of certain coral species, suggesting that there is a small number of stable members (i.e., the “coral microbial core”; Ainsworth et al., 2015; Hernandez-Agreda et al., 2017). Thus, it has been proposed that the coral bacterial community can be partitioned into a stable core component, a site-specific component, and a dynamic and variable component highly influenced by changes in abiotic and biotic factors (Hernandez-Agreda et al., 2016; Leite et al., 2018).

Recent advances in our understanding of the response of microbial community composition to environmental change have highlighted the potential role microbes play in coral host resilience. For instance, a stable microbiome has been suggested to confer host resilience when exposed to extreme conditions (O’Brien et al., 2018). Conversely, the microbiome has also been found to change and recover according to rapid environmental shifts (e.g., tides; Sweet et al., 2017 or acute pollution run-off events; Garren et al., 2009). Environmentally driven changes in the microbiome that result in increases or incorporation of better-adapted microbial taxa could theoretically aid or improve coral survival (e.g., Reshef et al., 2006; van Oppen et al., 2015, 2017; Peixoto et al., 2017; Torda et al., 2017). Understanding natural variability of the coral microbiome, particularly the potential for cyclical seasonal variation, can provide insight into how the microbiome may respond to environmental fluctuations and climate warming. Seasons present natural changes in factors such as temperature and irradiance (Warner et al., 2002; Bahr et al., 2017), dissolved calcium carbonate levels and aragonite saturation rate (Bates et al., 2010), and nutrient levels (particularly for coastal reefs influenced by run-off during rainy seasons; Costa et al., 2006). Long-term studies are available for Symbiodiniaceae communities, which have identified that some coral species exhibit seasonal variation in community composition (e.g., Chen et al., 2005; Ulstrup et al., 2008) while others remain stable through time (e.g., Thornhill et al., 2006a,b). Seasonal changes in Symbiodiniaceae may also manifest as changes to cell density, pigmentation or photo-efficiency (Fitt et al., 2000; Warner et al., 2002; Ulstrup et al., 2008). Variations in the coral bacterial community among time points have been suggested to reflect seasonal differences in their environment (e.g., Ceh et al., 2011; Kimes et al., 2013; Li et al., 2014; Sharp et al., 2017; Cai et al., 2018); however, all of these studies have lasted less than 1 year, which is insufficient to test hypotheses regarding seasonality. Indeed, one longer-term study (Yang et al., 2017) found the bacterial community of the brooding coral *Stylophora pistillata* to be dynamic, but not reflective of seasonal cycles (Yang et al., 2017).

The present study aimed to expand our understanding of long-term fluctuations in microbial community composition and examine whether temporal variations within the Symbiodiniaceae and bacterial members of the coral microbiome correlate with a seasonal cycle. To this aim, the community composition of bacteria and Symbiodiniaceae was characterized by DNA metabarcoding across a 2-year time period in two common species of coral on great barrier reef (GBR), *Acropora hyacinthus* and *Acropora spathulata.* Seasonality as well as the co-occurrences of microbial taxa through time were examined.

## Materials and Methods

### Sample Collection and Processing

Twelve colonies each of two species of coral, *A. hyacinthus* and *A. spathulata*, at two mid-shelf reefs in the central GBR, Rib reef (18°29’4.8”S, 146°52’13.7”E), and Davies reef (18°49’23.8”S, 147°38’56.2”E), were tagged and sampled over a 2-year time period. Both reef locations are similar distance from land, 75 and 79 km offshore, respectively. Sampling sites were back reef lagoon patch reef habitats and colonies were tagged at similar depths between 3–6 m. Sampling took place in February (end of summer) and October/November (end of winter) of both 2014 and 2015, as well as an additional time point in April 2015, making a total of five time points (Supplementary Table S1). At each time point and each location, a small nubbin of each colony was collected and immediately snap-frozen in liquid nitrogen (LN_2_). Frozen samples were then freeze-dried and crushed using a hydraulic bench top laboratory press prior to DNA extraction. Average monthly temperature data over the duration of the study period from both Rib and Davies reef were obtained from publicly available data collected by the Australian Institute of Marine Science (AIMS Historical Data Tool^1^).

DNA was extracted using a traditional salting out method with an added lysozyme digestion and bead-beating step (Damjanovic et al., 2017). Amplification of double-stranded products from the 16S rRNA gene for bacteria and the internal transcribed spacer region 2 (ITS2) was achieved through polymerase chain reaction (PCR) using gene-specific primers. The V5-V6 region of 16S was targeted using the primers 784F 5′-TCGTCGGCAGCGTCAGATGTGTATAAGAGACAG AGGATT AGATACCCTGGTA-3′ and 1061R 5′-GTCTCGTGGGCTC GGAGATGTGTATAAGAGACAG CRRCACGAGCTGACGAC-3′ (Andersson et al., 2008). ITS2 was targeted using the primers ITS2F 5′-TCGTCGGCAGCGTCAGATGTGTATAAG AGACAG GTGAATTGCAGAACTCCGTG-3′ and ITS2R 5′-GTCTCGTGGGCTCGGAGATGTGTATAAGAGACAG CCTCC GCTTACTTATATGCTT-3′ (Pochon et al., 2012). Both sets of primers included the Illumina adapter overhangs for Illumina MiSeq sequencing, underlined in the above primer sequences.

The 16S PCR was carried out in triplicate 10 μL reaction volumes, resulting in 30 μL pooled PCR product. Each reaction consisted of: 5 μL of AmpliTaq Gold MasterMix (Applied Biosystems), 2 μL of each primer (2 μM stock), and 1 μL of DNA template, with an additional no template control to test for contamination. All 16S reactions were run on a Kyratec SC-200 thermal cycler (Kyratec Life Science) using the following protocol: initial denaturation at 95°C for 10 min, then 30 cycles of denaturation at 95°C for 30 s, annealing at 57°C for 60 s and extension at 72°C for 60 s, followed by a final extension at 72°C for 7 min. The ITS2 PCR was also carried out in triplicate 10 μL reactions. Each reaction consisted of 5 μL of Qiagen Mulitplex MasterMix (Qiagen), 3 uL Milli Q, 0.5 μL of each primer (4 uM stock), and 1 μL of DNA template. All ITS2 reactions were run on a Kyratec SC-200 thermal cycler (Kyratec Life Science) using the following protocol: 95°C for 5 min, then 31 cycles of denaturation at 95°C for 30 s, annealing at 55°C for 30 s, and extension at 72°C for 30 s, followed by a final extension at 72°C for 5 min. All PCR products were then examined using gel electrophoresis on a 2% TBE-agarose gel stained with Ethidium Bromide (EtBr). Some ITS2 products displayed double-banding, representing both the target and a mitochondrial band. The target bands were poked using the tip of a clean pipette, introduced to clean PCR master mix, and underwent a second PCR with the same specifications but only 12 cycles of denaturation, annealing, and extension. These products were again checked by gel electrophoresis to ensure no double-banding prior to sequencing. PCR clean-up, indexing and sequencing were carried out at the Ramaciotti Centre for Genomics at the University of New South Wales on a 2 bp × 300 bp Illumina MiSeq run. Data was returned as de-multiplexed paired-end sequences. Some coral samples had no amplification for either 16S or ITS2 and were thus left out of the downstream analyses (see Supplementary Table S2 for final sample sizes).

### Sequence Assembly, Quality Control, and Taxonomic Assignment

Demultiplexed sequences for both 16S and ITS2 were assembled, checked for quality and assigned taxonomic classification using a QIIME2 v 2017.10 pipeline with additional plug-ins (Bolyen et al., 2018). The plug-in demux (Bolyen et al., 2018) was used for visualizing read quality and setting quality filtering guidelines. Quality filtering, trimming of poor-quality bases, de-replication, chimera filtering, merging paired-end reads, and the identification of amplicon sequence variants (ASVs) were performed using the DADA2 plug-in (Callahan et al., 2016). For 16S, mitochondrial and chloroplast sequences were removed and taxonomy was assigned by training a naïve-Bayes classifier on the V5-V6 region of the 16S gene in the SILVA 128 database (Quast et al., 2013) using the feature-classifier plugin (Bolyen et al., 2018) to match the primers used. Due to the high number of single-variants found for ITS2 and the subsequent small taxonomic database, it was not useful to use a classifier as above because the resolution was too low. Therefore, these single-variants for ITS2 were clustered by 97% similarity using a vsearch plug-in (Rognes et al., 2016). Taxonomic assignment was done according to the database from Arif et al. (2014). This allowed assignment down to the sub-type level for Symbiodiniaceae. At the end of the pipelines for both 16S and ITS2, the taxa plug-in (Bolyen et al., 2018) was used to create a feature table (biom table), and a taxonomy table with raw sequence counts that could then be used for further downstream analyses.

### Statistical Analyses

Data were read into R v. 3.5.0 (R Core Team, 2018) and analyzed using the package phyloseq (McMurdie and Holmes, 2013). Contaminants and singletons were removed from the 16S and ITS2 datasets prior to further analyses. Contaminants were identified using a similar method to that outlined in Lee et al. (2015); as contaminant taxa are expected to have high relative abundance in negatives and low relative abundance in samples, any ASV that exhibited a relative abundance of one or more orders of magnitude higher in negatives compared with coral samples were removed. Variations in alpha diversity (Shannon diversity index) and observed species richness of both bacteria and Symbiodiniaceae from the two coral species at both reefs and among time points were analyzed by analysis of variance (ANOVA) using a linear model fit by restricted maximum likelihood (REML) for repeated measures with an added autoregressive 1st order (AR1) correlation structure to account for time series autocorrelation in the R packages car (Fox and Weisberg, 2011) and nlme (Pinheiro et al., 2018). *Post hoc* comparisons were made using Tukey’s test with the packages multcompView (Graves et al., 2015) and lsmeans (Lenth, 2016). Differences in beta-diversity among species, reefs, and time points were assessed using permutational multivariate analysis of variance (PERMANOVA) blocked by colony to account for repeated measures. Homogeneity of dispersions was assessed using PERMDISP. Both PERMANOVA and PERMDISP were run with 999 permutations and beta-diversity was visualized using NMDS fit with environmental variables through constrained correspondence analysis (CCA) using the function envfit in vegan (Oksanen et al., 2018). Further exploration of microbial communities were examined through visualizing relative abundances with ggplot2 (Wickham, 2009), and bacterial indicator taxa (i.e., taxa that are identified as indicative of a specified treatment using both presence/absence and relative abundance) were characterized for each coral species at each reef among repeated time points (i.e., February and October/November) using a multi-level pattern analysis with 999 permutations in the package indicspecies (De Cáceres and Legendre, 2009). These indicators are used to identify consistencies between repeated time points.

Co-occurrences between bacteria and Symbiodiniaceae were determined using Spearman Rank correlation coefficients on ASVs appearing at least once in 20% or more of the samples using the packages corrplot (Wei and Simko, 2017) and igraph (Csardi and Nepusz, 2006) to optimize the number of microbial taxa included in the correlations. Correlation matrices of each species per reef were visualized in corrplot v. 0.84 (Wei and Simko, 2017). Significant correlations (>0.6 and <−0.6, *p* < 0.05) were identified and visualized as networks for each time point using Cytoscape v. 3.6.1 (Shannon et al., 2003).

## Results

### Temperature at Rib and Davies Reefs

**FIGURE 1:**
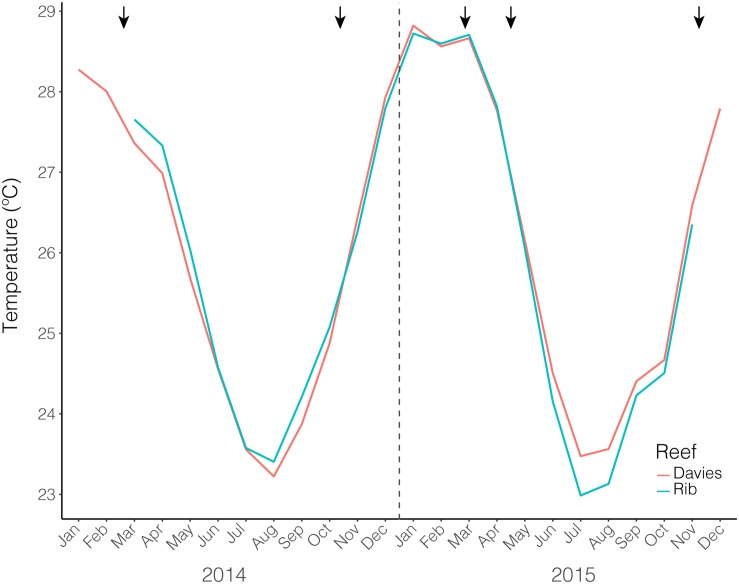
Average monthly temperatures from both Rib and Davies reef from January 2014 – December 2015. Black arrows indicate sampling time points.

The average monthly temperatures for both Rib and Davies reefs exhibited a seasonal pattern and were similar through time (Figure 1). The February time points were situated at the maximum of the annual temperature cycle, however, February 2015 was approximately 0.5°C warmer than February 2014 (February 2014 data only available for Davies reef). October/November time points were situated at the end of the winter season, just after temperatures had begun to increase. In contrast to the summer timepoints, October/November 2015 was between 0.4 and 0.5°C cooler than October/November 2014.

### Bacterial Community Characterization

A total of 5,112,489 sequences from 216 samples corresponding to 14,083 unique ASVs were recovered to characterize the bacterial communities of *A. hyacinthus* and *A. spathulata* at the two mid-shelf reefs through time. Negative controls were checked for contamination and three ASVs from the genera *Bradyrhizobium*, *Ralstonia*, and *Oxalobacteraceae* were removed from the dataset.

**FIGURE 2:**
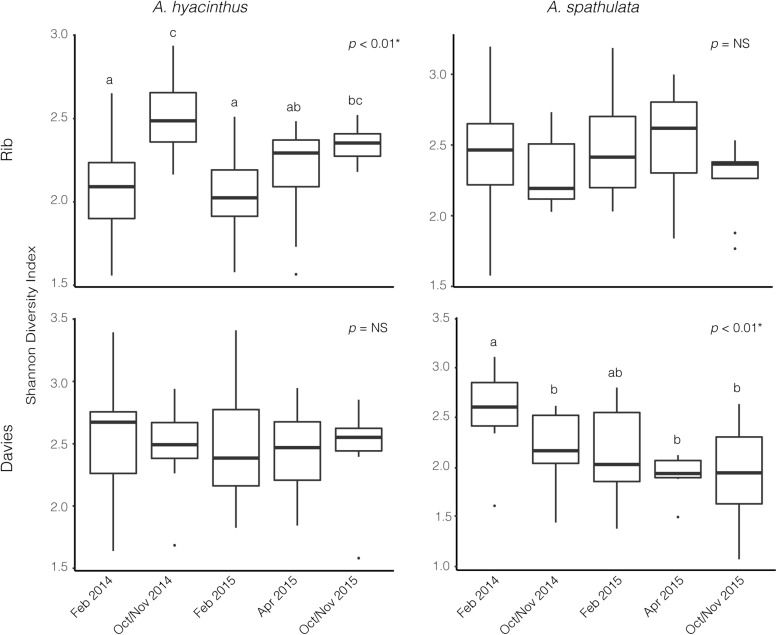
Alpha diversity based on the Shannon Diversity Index for bacterial communities through time for *A. hyacinthus* and *A. spathulata* at Rib and Davies reef.

Alpha diversity significantly varied through time for *A. hyacinthus* at Rib reef (ANOVA: df = 4, *F* = 7.39, *p* < 0.001) and for *A. spathulata* at Davies reef (ANOVA: df = 4, *F* = 4.75, *p* < 0.01). Alpha diversity did not significantly vary over time for *A. hyacinthus* at Davies reef or *A. spathulata* at Rib reef (Figure 2). Observed richness through time was also inconsistent across species and reefs. On average, *A. hyacinthus* had an average richness of 44.21 ± 2.5 and 48.7 ± 2.9 at Rib and Davies reefs, respectively, and *A. spathulata* had an average of 42.94 ± 1.64 and 40.44 ± 1.63 at Rib and Davies, respectively. Richness significantly varied through time for *A. hyacinthus* at Rib reef (ANOVA: df = 4, *F* = 2.69, *p* < 0.05) and for *A. spathulata* at both Rib (ANOVA: df = 4, *F* = 2.79, *p* < 0.05) and Davies reefs (ANOVA: df = 4, *F* = 3.13, *p* < 0.05), but not for *A. hyacinthus* at Davies reef.

**FIGURE 3:**
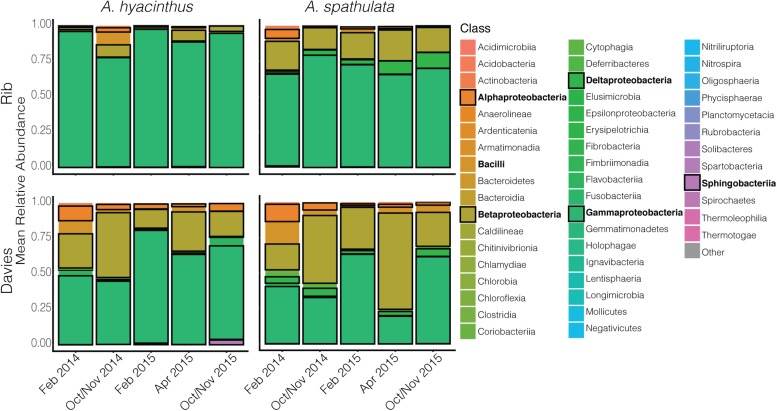
Mean relative abundance of bacterial classes through time for both *A. hyacinthus* and *A. spathulata* at Rib and Davies reef. Classes in bold represent those that are in highest relative abundance.

Bacterial communities of all samples were dominated by the classes Gammaproteobacteria, Betaproteobacteria, Bacilli, Alphaproteobacteria, and Deltaproteobacteria (Figure 3 and Supplementary Figure S1). Gammaproteobacteria, which was dominated by the genus *Endozoicomonas*, made up a higher percentage of the community in samples taken from Rib reef, where it made up 90.2 ± 2.2% and 70.3 ± 2.9% (mean ± SEM) for *A. hyacinthus* and *A. spathulata*, respectively. At Davies reef, Gammaproteobacteria accounted for 60 ± 3.5% for *A. hyacinthus* and 45.4 ± 3.9% for *A. spathulata*. An opposing pattern was observed for Betaproteobacteria; this class, which was dominated by the genus *Burkholderia-Paraburkholderia*, made up a higher percentage in samples taken at Davies reef as opposed to Rib reef. Betaproteobacteria at Davies reef accounted for 26.2 ± 2.9% and 34.3 ± 3.3% of the bacterial communities for *A. hyacinthus* and *A. spathulata*, respectively. At Rib reef, Betaproteobacteria made up only 4.54 ± 1.1% and 18.6 ± 2.02% of the communities for *A. hyacinthus* and *A. spathulata*, respectively.

**FIGURE 4:**
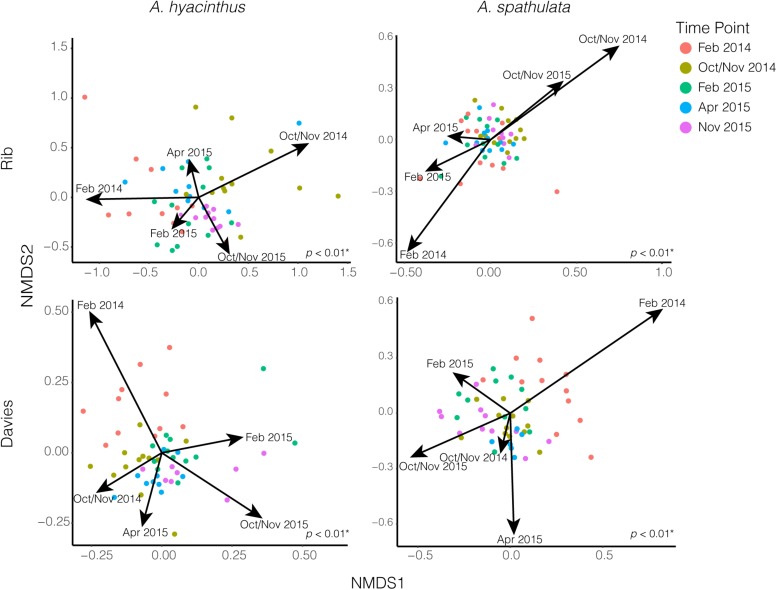
NMDS plots of bacterial communities for both *A. hyacinthus* and *A. spathulata* at Rib and Davies reef. Arrows representing time point were fit and scaled by constrained correspondence analysis (CCA). *p*-values in the lower right-hand corner of each nMDS plot represent PERMANOVA results.

Beta-diversity significantly varied according to reef, species and time point (Supplementary Table S3). Due to significant interactions, beta-diversity was also examined for each species at each reef where it significantly varied by time point; *A. hyacinthus* at Rib reef (PERMANOVA: df = 4, *F* = 3.97, *p* < 0.01), *A. hyacinthus* at Davies reef (PERMANOVA: df = 4, *F* = 3.16, *p* < 0.01), *A. spathulata* at Rib reef (PERMANOVA: df = 4, *F* = 1.45, *p* < 0.01) and *A. spathulata* at Davies reef (PERMANOVA: df = 4, *F* = 2.83, *p* < 0.01). Pairwise PERMANOVA suggested no significant differences in the bacterial communities of *A. hyacinthus* among repeated sampling time points in February for Rib reef, but there were significant differences between the two February time points at Davies reef and between the October/November time points at both reefs (Supplementary Table S4). Pairwise PERMANOVA suggested no significant differences in bacterial communities of *A. spathulata* for all repeated sampling time points (February 2014 vs. February 2015 and October/November 2014 vs. October/November 2015) at Rib reef, but did show significant variations between repeated sampling points in February at Davies reef (Supplementary Table S5). For *A. hyacinthus* at both Rib and Davies reef the two February time points did not differ significantly, but the two October/November time points did (Supplementary Table S6). These data are supported by the CCA fitted time point vectors in nMDS, where data clouds from repeated sampling time points did not pull in the same direction, except for *A. spathulata* at Rib reef, where October/November 2014 and October/November 2015 both pulled in the same direction (Figure 4). According to CCA, time point represented a significant proportion (*p* < 0.05) of variation for both coral species and both reefs; 34 and 42% for *A. hyacinthus* at Rib and Davies reefs, respectively, and 14 and 39% for *A. spathulata* at Rib and Davies reefs, respectively.

### Indicator Taxa for Repeated Sampling Time Points

No significant bacterial indicator taxa were identified for *A. hyacinthus* at Rib reef for February, and neither coral species at either reef showed any significant bacterial indicators for October/November time points. Two indicator taxa of the February time points were recovered for *A. hyacinthus* at Davies reef: one *Endozoicomonas* ASV and one *Pseudoalteromonas*. Three indicator taxa of the February time points were identified for *A. spathulata* at Davies reef, including two *Endozoicomonas* ASVs, one of which was the same ASV as found in *A. spathulata* at Davies reef, and one *Vibrio.* One *Endozoicomonas* ASV, which was different from the others, was found as the single indicator taxon of the February time points for *A. spathulata* at Rib reef (see Supplementary Table S7).

### Symbiodiniaceae Community Characterization

**FIGURE 5:**
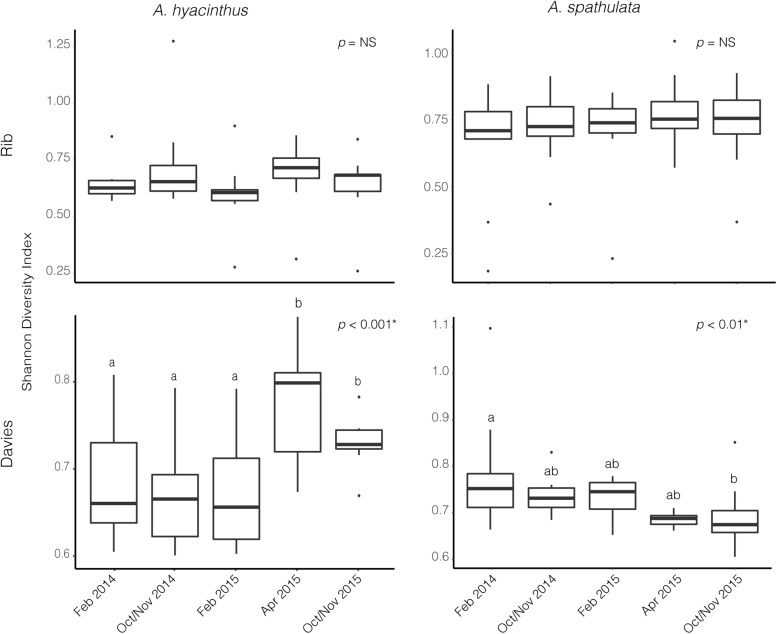
Alpha diversity based on the Shannon Diversity index for Symbiodiniaceae communities through time for both *A. hyacinthus* and *A. spathulata* at Rib and Davies reef.

A total of 6,418,776 sequences from 215 samples corresponding to 54 unique Symbiodiniaceae sequence types were recovered to characterize the Symbiodiniaceae communities of both *A. hyacinthus* and *A. spathulata* at the two reefs and across the five time points. Alpha diversity of Symbiodiniaceae remained stable through time for *A. hyacinthus* and *A. spathulata* at Rib reef, but significantly varied for both species at Davies reef (ANOVA_hyacinthus_: df = 4, *F* = 16.36, *p* < 0.001; ANOVA_spathulata_: df = 4, *F* = 4.07, *p* < 0.01; Figure 5). Observed species richness remained stable for *A. hyacinthus* and *A. spathulata* at both Rib and Davies reefs among time points.

**FIGURE 6:**
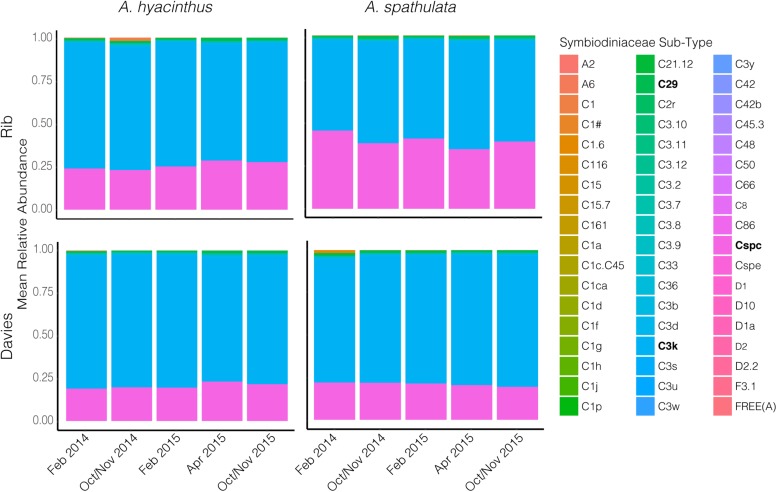
Mean relative abundance of Symbiodiniaceae sequence sub-types through time for both *A. hyacinthus* and *A. spathulata* at Rib and Davies reef.

*Cladocopium* C3k and Cspc sequence types were the dominant Symbiodiniaceae taxa through time for both species at both reefs (Figure 6 and Supplementary Figure S2) making up an average of 69.3 ± 1.2% and 26.8 ± 1.2% (mean ± SEM), respectively, of the Symbiodiniaceae communities harbored by *A. hyacinthus* and *A. spathulata*. Beta-diversity also remained stable through time for *A. hyacinthus* at Rib and Davies Reef and for *A. spathulata* at Rib Reef. Beta-diversity of *A. spathulata* at Davies reef significantly varied among time points (PERMANOVA: df = 4, *F* = 4.86, *p* < 0.01; Supplementary Table S8), driven only by community differences between the two time points February 2015 and October/November 2015. Dominant taxa remained the same between these two time points, but the colonies had incorporated a low background abundance of the *Cladocopium* C1d type in February 2015, while in October/November 2015 these same colonies had replaced C1d with low background abundances of *Cladocopium* C3 types, including C3.10 and C3.12.

### Co-occurrences of Bacterial and Symbiodiniaceae Taxa

**FIGURE 7:**
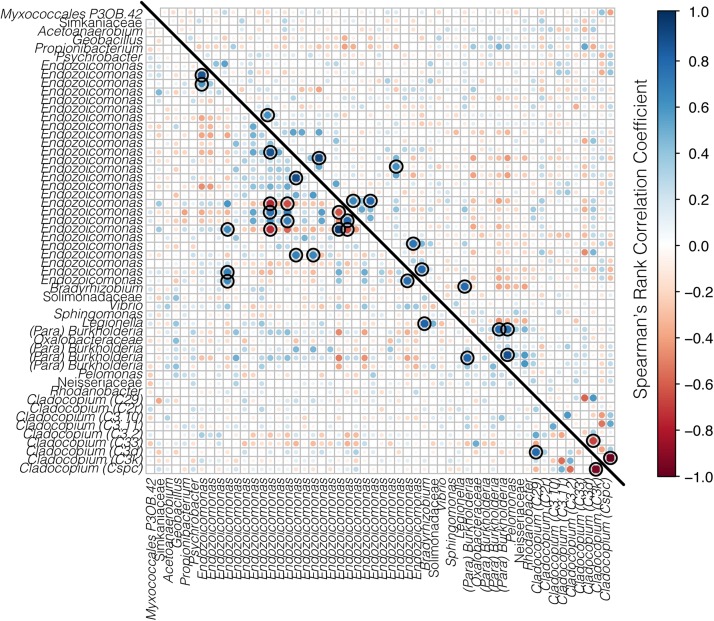
Correlation matrix of both bacteria and Symbiodiniaceae for *A. hyacinthus* from Rib reef (below the diagonal line) and Davies reef (above the diagonal line). Bacterial taxa represent ASVs classified to genus where possible, and Symbiodiniaceae are represented by genus and sub-type. Positive correlations are represented in blue and negative correlations are represented in red, where both the size and the color of the dots represent the strength of the correlation. Black rings represent significant positive or negative correlations (*p* < 0.05).

**FIGURE 8:**
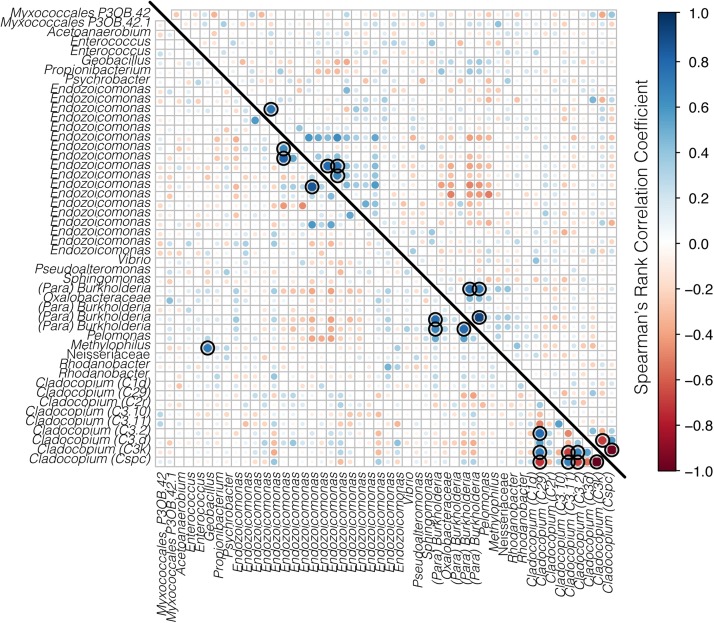
Correlation matrix of both bacteria and Symbiodiniaceae for *A. spathulata* from Rib (below the diagonal line) and Davies reef (above the diagonal line). Bacterial taxa represent ASVs classified to genus where possible, and Symbiodiniaceae are represented by genus and sub-type. Positive correlations are represented in blue and negative correlations are represented in red, where both the size and the color of the dots represent the strength of the correlation. Black rings represent significant positive or negative correlations (*p* < 0.05).

The co-occurrences and correlation strengths of bacterial and Symbiodiniaceae taxa differed among time points within species and reef (Supplementary Figure S3). Among time points, Symbiodiniaceae correlated both positively and negatively with a number of bacterial taxa, including *Endozoicomonas, Burkholderia-Paraburkholderia*, *Sphingomonas*, and others. However, only a small number of taxa had significant correlations (>0.6 or <−0.6, *p* < 0.05) when all time points were considered together for each species at each reef (Figures 7, 8). Symbiodiniaceae significantly correlated only with other Symbiodiniaceae, where the two dominant types, *Cladocopium* C3k and Cspc were negatively correlated with each other for both coral species at each reef. Most significant bacterial correlations occurred between ASVs of the same genus, for instance *Endozoicomonas* with *Endozoicomonas* and *Burkholderia-Paraburkholderia* with *Burkholderia-Paraburkholderia*. Some *Endozoicomonas* ASVs correlated positively with each other, while others correlated negatively.

## Discussion

### Microbial Communities Were Temporally Variable, but Do Not Exhibit a Seasonal Pattern

The bacterial communities of both *A. hyacinthus* and *A. spathulata* were dominated by Proteobacteria, including the classes Gammaproteobacteria, Betaproteobacteria and Alphaproteobacteria, a pattern commonly observed in *Acropora* species (e.g., Ceh et al., 2011; Littman et al., 2011; Meron et al., 2011; Ziegler et al., 2017). The overall community composition was similar between *A. hyacinthus* and *A. spathulata* at the two reefs with little difference among colonies (i.e., genotype; Glasl et al., 2019), but there were consistent differences in the relative abundance of certain taxa such as the Gamma- and Betaproteobacteria according to reef. Coral bacterial communities have previously been found to have strong location or geographic signatures (e.g., Littman et al., 2009; Leite et al., 2018), and it has been proposed that the coral microbiome includes a site or location-specific component (Hernandez-Agreda et al., 2016). Rib and Davies reef are mid-shelf reefs on the GBR, both influenced by oceanic inflow (Brinkman et al., 2002). However, they are located approximately 150 km apart and could have different environmental influences, including differences in hydrodynamic flow, upwelling from offshore and land-based influences, such as nutrient levels as a result of run-off (e.g., Davies reef is much closer to the mouth of the Burdekin River than Rib reef, which can have extensive flood plumes; Wolanski and Van Senden, 1983). This may result in more variability in the environmental pool of microbes.

While there were significant variations in bacterial communities through time, there was little evidence of seasonal patterns within species and across reefs. Previous studies on the coral microbiome have also identified significant differences in the bacterial community composition through time (e.g., Ceh et al., 2011; Kimes et al., 2013; Li et al., 2014; Sharp et al., 2017; Cai et al., 2018). These studies were completed within a 1-year time period, yet suggested the observed changes were seasonal. The pattern from the present study instead supports the findings by Yang et al. (2017), where *S. pistillata* also exhibited highly dynamic temporal variations in its bacterial community composition, but with little evidence of seasonal cycles over a 2-year period. Therefore, the variations in bacterial communities among time points are likely influenced by factors other than season. This reinforces the conclusions made by Yang et al. (2017), who recommend that studies with a duration longer than 1 year are essential for understanding the drivers of temporal change in bacterial communities. Perhaps studies that exceed even 2 years are necessary to find consistent cyclical patterns due to inter-annual variation in environmental parameters and to identify potential ontogenetic microbial shifts (e.g., Williams et al., 2015).

The dominant Symbiodiniaceae sequence types in *A. hyacinthus* and *A. spathulata* included both *Cladocopium* C3k and *Cladocopium* Cspc. *Cladocopium* C3 has been identified as a common symbiont of acroporids from central and southern GBR reefs (e.g., LaJeunesse et al., 2003, 2004b). In the present study, *Cladocopium* Cspc co-dominated, but was negatively correlated with C3k, perhaps representing a competitive interaction between the two dominant strains. However, these dominant strains were maintained throughout the 2-year sampling period, suggesting overall community structure was stable and did not reflect seasonal variation. Seasonal variation in the dominant Symbiodiniaceae types has been previously identified for some coral species (e.g., Chen et al., 2005; Ulstrup et al., 2008). In cases of temporally stable Symbiodiniaceae community composition (e.g., LaJeunesse et al., 2005; Thornhill et al., 2006a,b; Klepac et al., 2015; Cai et al., 2018), other seasonal variations are often present such as changes to cell density, pigment content, or photosynthetic efficiency (Fitt et al., 2000; Warner et al., 2002; Ulstrup et al., 2008). Seasonal variation in overall community composition was not evident in the data presented here, and smaller changes among time points were driven by background types, particularly at Davies reef, where alpha diversity of both *A. hyacinthus* and *A. spathulata*, and beta-diversity of *A. spathulata* varied significantly. Spatially, the population structure of *Cladocopium* spp. symbionts can be more complex than their hosts, showing divergence among closely located reefs (Davies et al., 2019), and suggesting there may have been different environmental pools of *Cladocopium*-type symbionts at the two reefs that were available for corals to uptake. Further, *A. spathulata* at Davies reef showed the acquisition of novel background algal strains among time points. This symbiont switching in the rare biosphere has been found previously, but until recently, only following considerable bleaching events (Lewis and Coffroth, 2004; Boulotte et al., 2016) that did not occur even during peak summer temperatures during the present study. In the present study, switching was species-specific, suggesting some level of host regulation and supporting the idea of a host-specific “Symbiodiniaceae signature,” as outlined in Rouzé et al. (2019).

Correlation and network analyses are often used to examine interactions between members of the coral holobiont. However, the majority of network studies in corals have so far looked at a single taxonomic group (e.g., bacteria: reviewed in Sweet and Bulling, 2017 or Symbiodiniaceae: Ziegler et al., 2018), while only a few have examined multiple groups (e.g., phage-bacteria: Soffer et al., 2015 or Symbiodiniaceae-bacteria: Bernasconi et al., 2018; Bonthond et al., 2018). In the present study, the small number of significant correlations found between microbial taxa for each coral species at each reef suggests that there were few co-occurrences or mutual exclusions (e.g., negative interactions) that were either persistent through time or following seasonal patterns. When time points were pooled, Symbiodiniaceae had no significant correlations with any bacterial taxa. When time points were analyzed separately, the bacterial communities had higher numbers of significant correlations, which resulted in complex networks, while Symbiodiniaceae were interconnected with both other Symbiodiniaceae and a small number of bacterial taxa. These networks, however, were not consistent through time, and did not display any obvious seasonal patterns of microbial interactions. Previous studies have found Symbiodiniaceae correlating only with other Symbiodiniaceae (e.g., Bonthond et al., 2018), while others have found some connectivity with bacterial taxa (e.g., Bernasconi et al., 2018). However, the low number of studies incorporating network analyses for both the Symbiodiniaceae and bacterial components of the coral microbiome currently limits the ability to assess common patterns. Additionally, more useful networks may be created in future studies with the incorporation of bacterial/algal functional or metabolic data rather than simply taxonomic identity (e.g., Zhou et al., 2010; Steinway et al., 2015).

The results from this study suggest that both the composition of bacteria and Symbiodiniaceae communities and the interactions among microbial taxa are dynamic through time; few interactions remained consistent among all time points in each species at each reef. Future microbial functional analyses will be necessary for understanding this variability, along with a better understanding of the regulation of microbial symbioses by the coral host. Our results suggest that caution should be taken when making conclusions from a network or correlation analysis that represents only a single time point.

### Potential for Functional Differentiation in *Endozoicomonas*

The present study found no bacterial indicator species that were significantly associated with the November time points across coral species. However, a small number of indicator taxa were consistently associated with both February time points for the two coral species, mostly consisting of *Endozoicomonas* strains. *Endozoicomonas* is a common bacterial genus that associates with a wide range of coral species, including those from the families Acroporidae (Ziegler et al., 2016, 2017), Pocilloporidae (Bayer et al., 2013; Neave et al., 2017a; van Oppen et al., 2018), Fungiidae (Roder et al., 2015), and Poritidae (Apprill et al., 2016). This genus has been suggested to play a number of functional roles linked with coral health, such as carbohydrate cycling and protein transport (Neave et al., 2016), dimethylsufiopropionate (DMSP) degradation (Bourne et al., 2013), provision of amino acids (Neave et al., 2016), and, importantly, thermal or bleaching protection (Pantos et al., 2015). The presence of an *Endozoicomonas* ASV as an indicator of the February time points may suggest this strain provides some benefits during warm summer temperatures.

The two coral species in the present study shared one *Endozoicomonas* ASV as an indicator, but were also populated with other *Endozoicomonas* ASVs that were not shared. This suggests some functional specificity among *Endozoicomonas* strains in relation to host species. Thus, while seasonal variation in overall community composition (i.e., taxonomy) was not observed, small differences in the relative abundance or presence/absence of closely related bacterial strains were observed, which may have consequences for the functional potential of these bacterial communities (e.g., Neave et al., 2017a). Interestingly, both the correlation and network analyses found that different *Endozoicomonas* strains correlated both positively and negatively with each other. While the correlation between different *Endozoicomonas* strains could be a result of intragenomic variation (*Endozoicomonas* genomes have been found to host more than one copy of the 16S rRNA gene depending on species; Neave et al., 2014), it may also imply functional differentiation among *Endozoicomonas* sequence variants (Neave et al., 2017b). This highlights the importance of examining higher resolution taxonomic classification (e.g., ASVs) in metabarcoding studies, and future studies should incorporate functional analyses.

### Conclusion

The microbiomes of *A. hyacinthus* and *A. spathulata* were complex and dynamic through time and variable according to reef and host species, yet not reflective of seasonality. This validates the findings of Yang et al. (2017) and reinforces their conclusion that long-term microbial surveys are essential for understanding the variable nature of the coral microbiome through time. Further studies are necessary to determine the consistency of these findings across greater time frames, spatial scales and coral taxa.

## Data Availability

The datasets generated for this study can be found in NCBI Sequence Read Archive, accession number: PRJNA491379.

## Ethics Statement

Anthozoans are not subject to ethics approval. Sampling for this study was carried out under the Great Barrier Reef Marine Park Authority and the Queensland Parks and Wildlife Service permit number G12/35236.1.

## Author Contributions

HE, HS, GT, and MO developed the research question. VM and NC were responsible for the original sampling design and both funding for and provision of the coral samples. HE performed the genetic laboratory work, bioinformatics, and analyses with statistical assistance from HS. HE was responsible for the initial draft of the manuscript, with editorial support from all co-authors.

## Conflict of Interest Statement

The authors declare that the research was conducted in the absence of any commercial or financial relationships that could be construed as a potential conflict of interest.

## Footnotes

1 http://data.aims.gov.au/aimsrtds/datatool.xhtml
