# Supplementary figures and images for: Temporal Variation in the Microbiome of Acropora Coral Species Does Not Reflect Seasonality
Source: Front Microbiol. 2019 Aug 16;10:1775. doi: 10.3389/fmicb.2019.01775 (PMC6706759; doi:10.3389/fmicb.2019.01775)

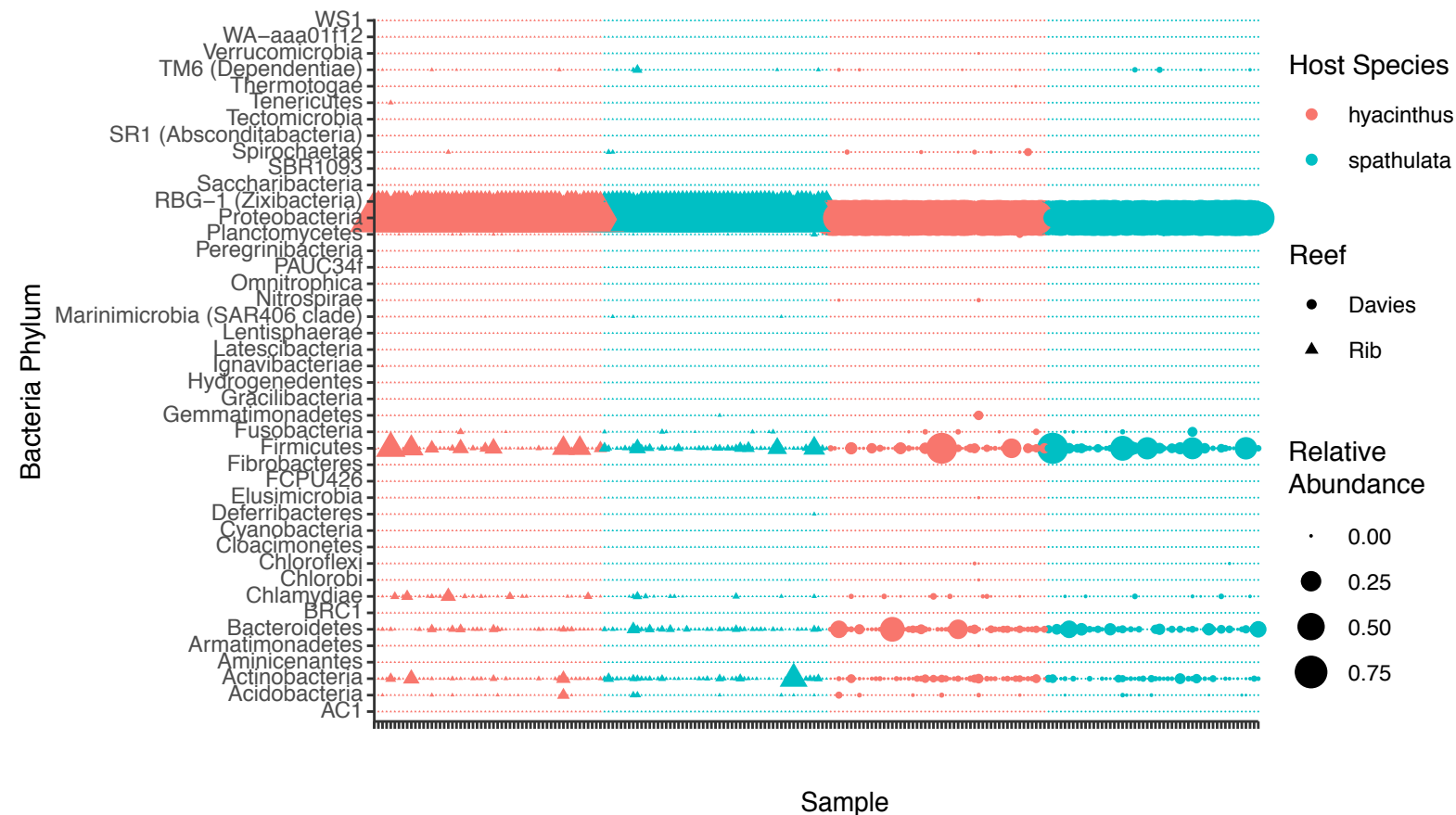

**Figure S1:** Relative abundance of bacteria phyla in each sample grouped by host species and reef.

Supplement: Supplementary file 1 [file Data_Sheet_1.PDF]
