# Supplementary material for: Temporal Variation in the Microbiome of Acropora Coral Species Does Not Reflect Seasonality
Source: Front Microbiol. 2019 Aug 16;10:1775. doi: 10.3389/fmicb.2019.01775 (PMC6706759; doi:10.3389/fmicb.2019.01775)
Supplement: Supplementary file 2 [file Data_Sheet_2.PDF]

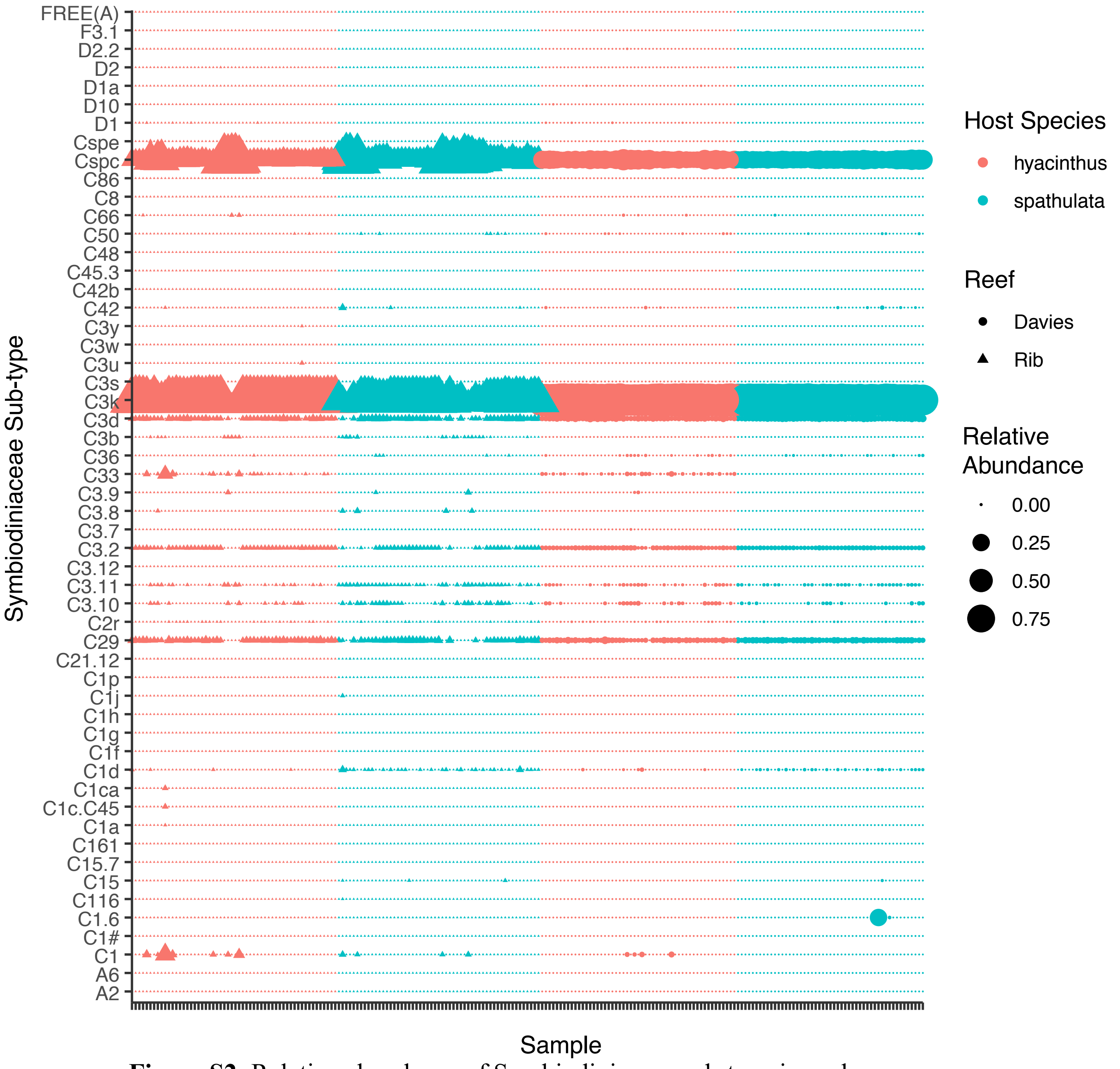

Figure S2: Relative abundance of Symbiodiniaceae sub-type in each sample grouped by host species and reef.
