# Supplementary material for: Temporal Variation in the Microbiome of Acropora Coral Species Does Not Reflect Seasonality
Source: Front Microbiol. 2019 Aug 16;10:1775. doi: 10.3389/fmicb.2019.01775 (PMC6706759; doi:10.3389/fmicb.2019.01775)
Supplement: Supplementary file 3 [file Data_Sheet_3.PDF]

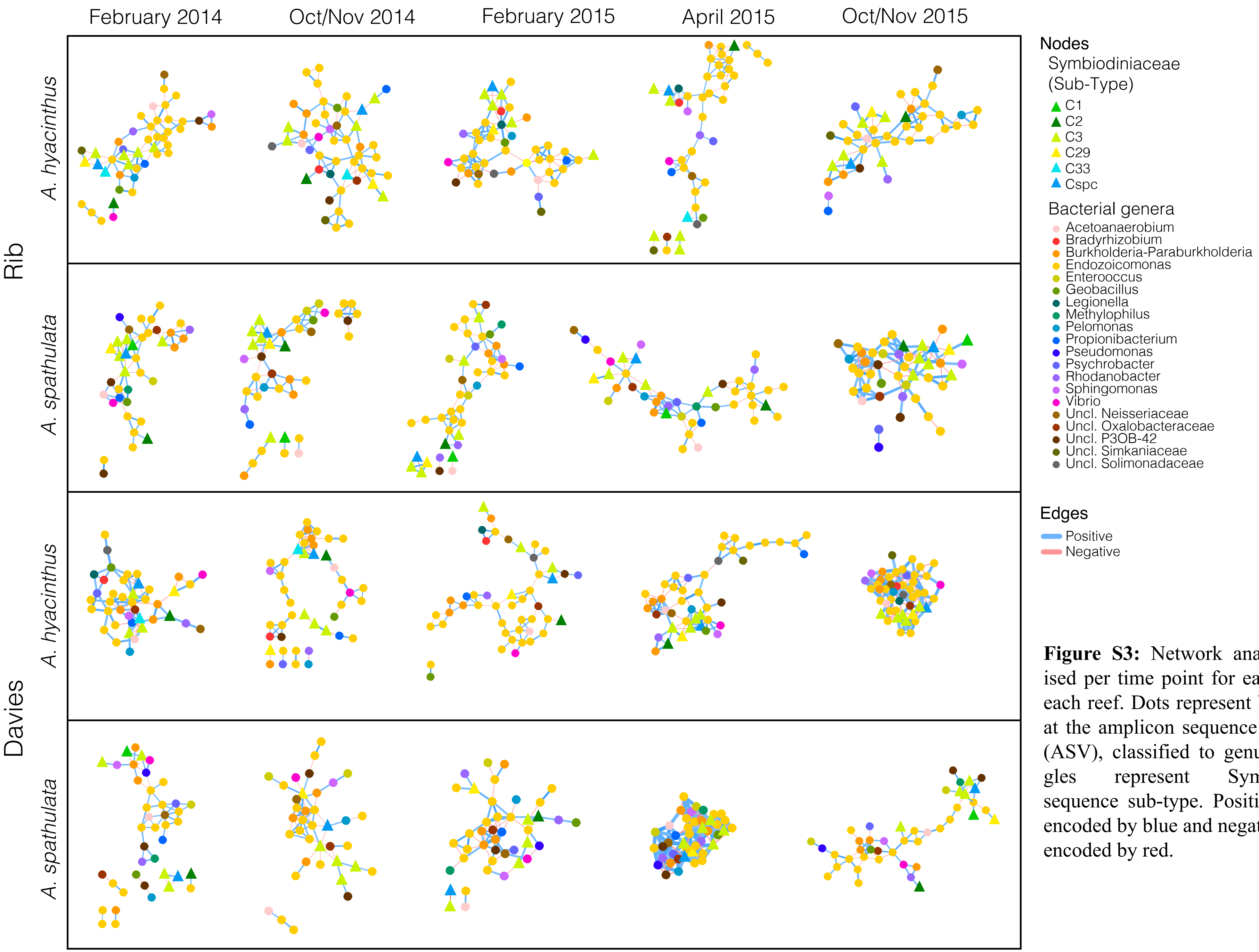

Figure S3: Network analyses visualised per time point for each species at each reef. Dots represent bacterial taxa at the amplicon sequence variant level (ASV), classified to genus, and triangles represent Symbidoiniaceae sequence sub-type. Positive edges are encoded by blue and negative edges are encoded by red.
